# Mechano‐YAP/TAZ‐regulated smooth muscle cells are an important source of Wnt signalling for gut regeneration

**DOI:** 10.1002/ctm2.70005

**Published:** 2024-08-16

**Authors:** Mintao Ji, Shuai Dong, Shuangshuang Lu, Haisheng Liang, Yiping Lin, Chenyu Luo, Haimeng Zheng, Yinyin Shu, Zhisen Zhang, Xiaoni Jin, Yuhan Guo, Kai Kang, Hong Zhang, Yuhong Wang, Hanna Lucie Sladitschek‐Martens, Sha Huang, Xiaobing Fu, Guangming Zhou, Zhenke Wen, Lei Chang

**Affiliations:** ^1^ State Key Laboratory of Radiation Medicine and Protection, School of Radiation Medicine and Protection The Fourth Affiliated Hospital of Soochow University, Collaborative Innovation Center of Radiation Medicine of Jiangsu Higher Education Institutions, Medical College of Soochow University Suzhou China; ^2^ The First Affiliated Hospital of Soochow University Suzhou China; ^3^ Department of Biochemistry and Biophysics University of California San Francisco California USA; ^4^ Key Laboratory of Tissue Repair and Regeneration of PLA The General Hospital of PLA Beijing China; ^5^ Jiangsu Key Laboratory of Infection and Immunity Institutes of Biology and Medical Sciences, Soochow University Suzhou China; ^6^ Institute of Radiation Medicine Shanghai Medical College, Fudan University Shanghai China

Dear Editor,

Inflammatory bowel disease (IBD) affects 8 million patients and cannot be completely cured.^1^ Hence, there is an unprecedented need to explore new treatment strategies for gut regeneration. Recent studies have identified several subepithelial cells that are involved in regulating intestinal stem cells through Wnt signalling.^2^, ^3^ Despite these advancements, our understanding of cellular communication and origins of Wnt signalling driving injured gut regeneration remains somewhat restricted. In this study, we utilised male genetic models and scRNA‐seq data to discover that mechano‐regulated yes‐associated protein (YAP) and the transcriptional coactivator with PDZ‐binding motif (TAZ) transcriptionally regulated WNT4 in smooth muscle cells (SMCs) to activate Wnt signalling in transit amplifying (TA) cells and promote injured gut regeneration.

Previous studies have indicated the pivotal role of mechanotransduction in tissue homeostasis.^4^, ^5^ To explore whether mechanotransduction undergoes changes in the context of IBD, we conducted a re‐analysis of RNA‐seq data from the 2490 endoscopy samples based on disease activity.^6^ Intriguingly, the contraction score exhibited a decline as disease activity increased, encompassing genes associated with contraction, such as PDE5A, MYL5, MYLK and CAMK2D. Simultaneously, the relaxation score increased with heightened disease activity, encompassing genes linked to relaxation, such as ADM, RAMP3, NOS2 and NOS3 (Figure 1A). Next, to examine whether mechanotransduction treatments could influence gut regeneration, the mice were administered with dextran sodium sulphate (DSS, 3.5%) for 5 days, in conjunction with the mechanotransduction‐related drugs, thereby simulating conditions of high (Norepinephrine [Nopp] and Vasopressin [Vaso]) or low (Tadalafil [Tada] and Icariin [Icar]) mechanical inputs (Figure 1B). Interestingly, mechano‐stimulatory drugs, such as Nopp and Vaso, significantly reduced the levels of CXCL1 and IL1β, while low mechanical drugs, such as Tada and Icar, had the opposite effects (Figure 1C,D). Additionally, hematoxylin‐eosin staining (HE), periodic acid‐schiff staining (PAS), KI67 and CL.CASPASE3 results corroborated these findings (Figure 1E–H), suggesting that high mechanical treatments facilitated the regeneration of injured gut, while low mechanical treatments exacerbated gut injury.

**FIGURE 1:**
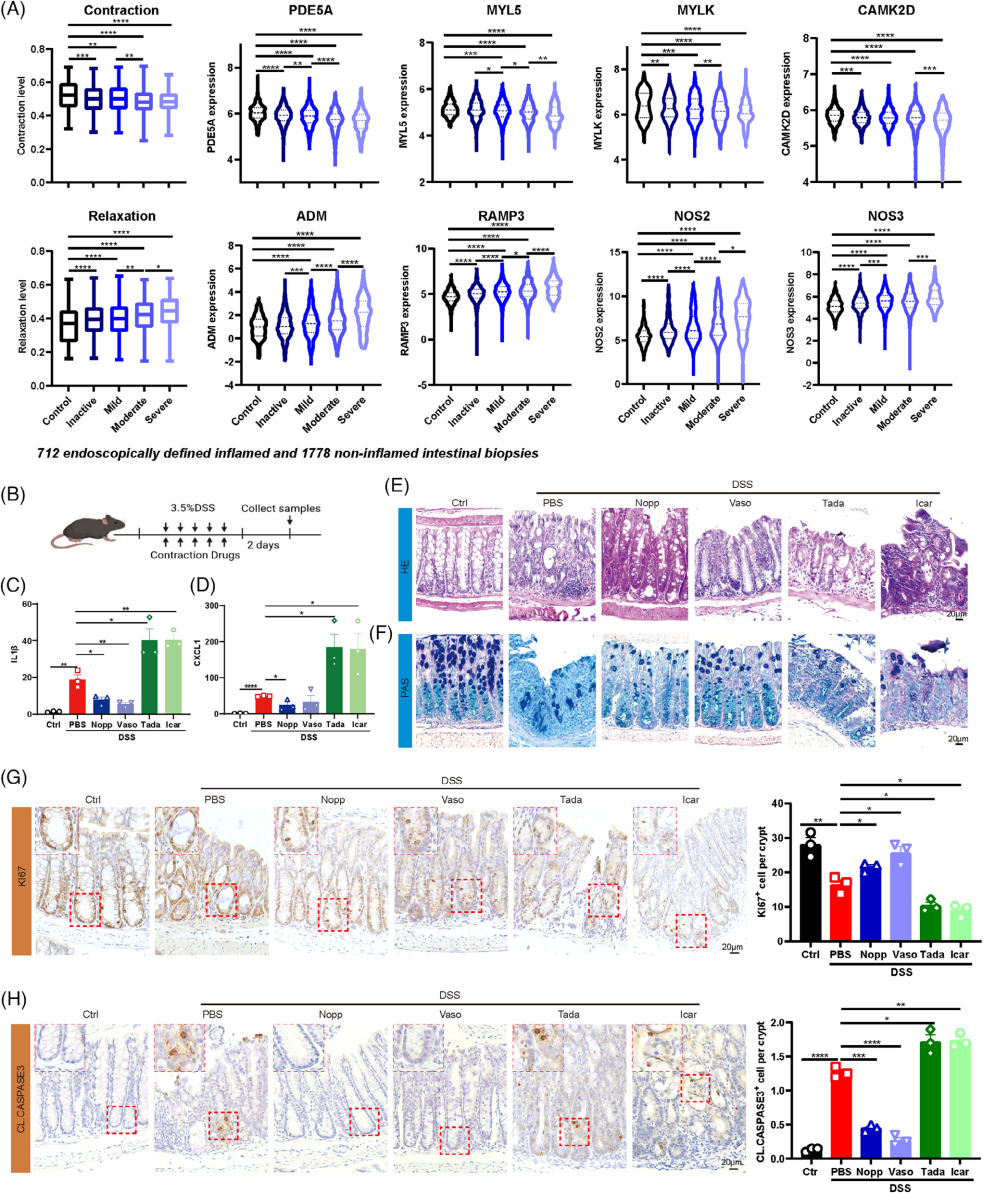
Alterations in mechanotransduction impact gut regeneration. (A) The levels of contraction signalling pathway and the contraction‐related genes (PDE5A, MYL5, MYLK and CAMK2D) in different stages of adult inflammatory bowel disease (IBD) patients and controls (upper). The levels of relaxation signalling pathway and the relaxation‐related genes (ADM, RAMP3, NOS2 and NOS3) in different stages of adult IBD patients and controls (bottom). (B) Schematic illustration of the experimental workflow of dextran sodium sulphate (DSS) and contraction drugs treatment. Expression of CXCL1 (C) and IL1β (D) in DSS and contraction drugs (Nopp [5 mM/kg per day, i.p.] and Vaso [.3 µg/kg per day, i.p.] are pro‐contraction drugs, Tada [10 mg/kg per day, i.p.] and Icar [10 mg/kg per day, i.p.] are pro‐relaxation drugs) treatment. Representative colon HE (E) images and PAS (F) images in DSS and contraction drugs treatment. (G) Representative image (left) and quantification (right) of colon KI67 immunohistochemistry in DSS and contraction drugs. (H) Representative image (left) and quantification (right) of colon CL.CASPASE3 immunohistochemistry in DSS and contraction drugs. Box plots indicate median (middle line), 25th, 75th percentile (box), minima, maxima. Violin plots indicate median (middle line), 25th, 75th percentile, minima, maxima. Bar charts are presented as the mean ± SEM. The statistical analysis was calculated by two‐sides unpaired Student's *t*‐test, the confidence interval is 95%. The point represents a mouse or human sample. Each experiment was repeated three independent times with similar results. *p < .05, **p < .01, ***p < .001 and ****p < .0001. Scale bars, 20 µm.

To investigate the microenvironmental changes in the injured gut, we re‐analysed human (SCP259^7^) or mouse (GSE201723^8^) scRNA‐seq data, and identified similar cell clusters (Figures S1A–E and S2). Using Cellchat analysis tool, we observed elevated interaction of SMCs‐TA cells in injured group compared with control group, especially the WNT4‐FZD3‐LRP5/6 ligand–receptor pair (Figures 2A,B, S1F and S3A–D). Furthermore, the injured group exhibited a significant increase in Wnt signalling activity specifically within TA cells, compared to the control group (Figures S1G–I and S3E–N). To further investigate the role of WNT4, we added the recombined WNT4 (rWNT4) and found that rWNT4 significantly increased the intestinal and colon organoid size (Figures 2C and S4A,B). Meanwhile, we also knocked down the WNT4 in smooth muscle cells of mouse aortic vessels (MOVAS cells) and collected their supernatant. We found that knocking down WNT4 significantly decreased the intestinal and colon organoid area (Figures 2D–F and S4C,D). Meanwhile, the SMCs WNT4 knockout mice (*Myh11^CreERT2^; Wnt4^fl/fl^*) were treated with 3.5% DSS for 5 days. HE, PAS and KI67 results implied that SMCs WNT4 knockout exhibited more pronounced gut damage, compared to the DSS‐treated WT group (Figure 2G–J). Further, DSS‐treated SMCs WNT4 knockout mice exhibited higher levels of IL1β and CCL2 (Figure 2K), indicating the critical roles of SMCs’ WNT4 in gut regeneration.

**FIGURE 2:**
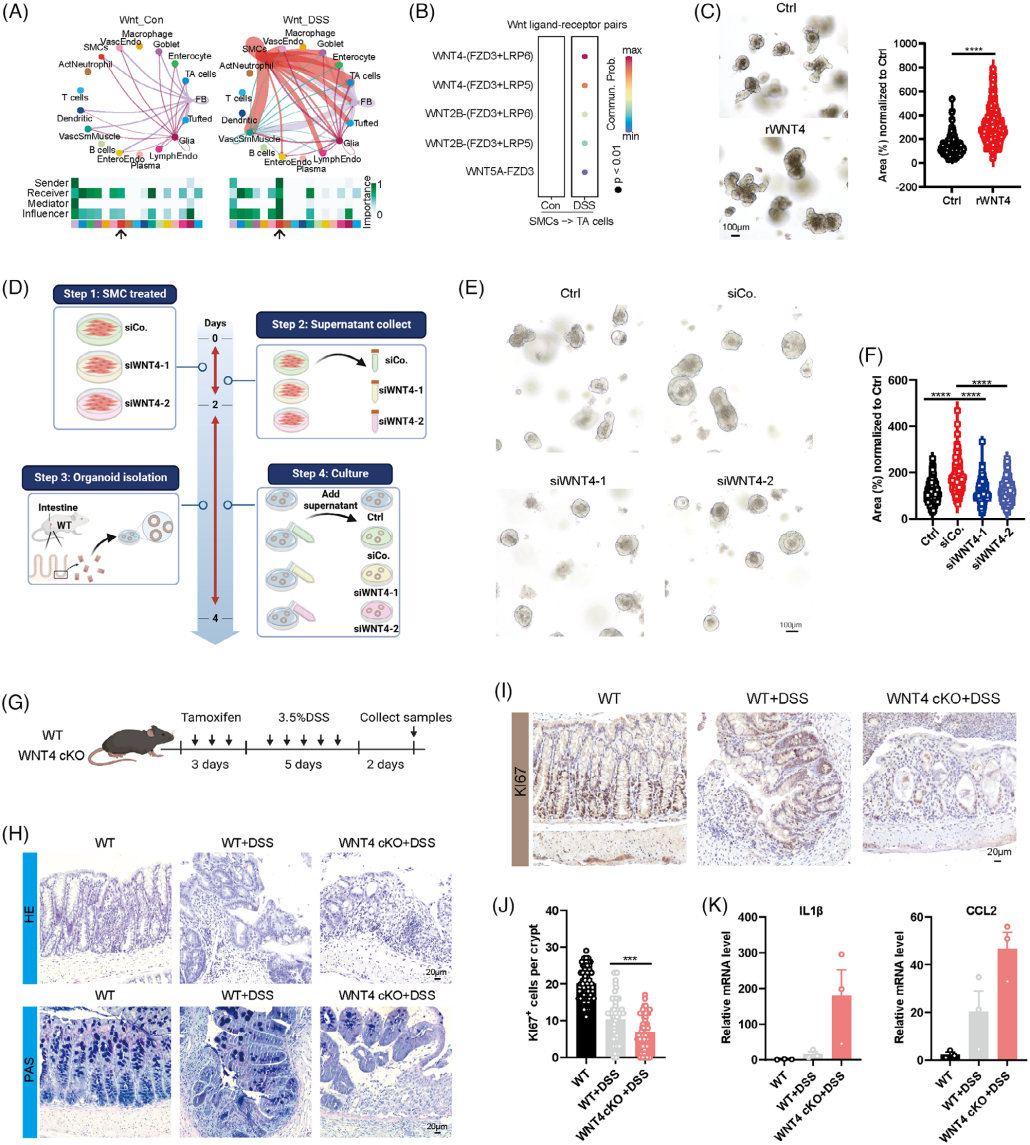
SMCs WNT4 knockout decreased the gut regeneration. (A) Inferred signalling networks for Wnt pathway in Con and dextran sodium sulphate (DSS) groups. Circle plots are shown on the top. Cell clusters that participate in signalling are annotated. Heatmaps showing the relative importance of each cell cluster as signal sender or receiver are shown at the bottom. (B) The levels of Wnt ligand–receptor genes in Con and DSS groups. (C) Representative (left) image and quantification (right) of intestinal organoid growth in control and rWnt4 groups for 3 days. (D) Schematic illustration of the experimental workflow of intestinal organoid culture. Representative (E) image and quantification (F) of intestinal organoid cultured with supernatant of control, siCo, siWNT4‐1 and siWNT4‐2 groups for 3 days. (G) Schematic illustration of the experimental workflow of DSS treatment. (H) Representative colon HE images (**upper**) and PAS images (bottom) from WT or WNT4 cKO mice treated with DSS. Representative image (I) and quantifications (J) of KI67 colon immunohistochemistry from WT or WNT4 cKO mice treated with DSS. (K) Expression of IL1β and CCL2 from WT or WNT4 cKO mice treated with DSS. Violin plots indicate median (middle line), 25th, 75th percentile, minima, maxima. Bar charts are presented as the mean ± SEM. The statistical analysis was calculated by two‐sides unpaired Student's *t*‐test, the confidence interval is 95%. The point represents a mouse or organoid sample. Each experiment was repeated three independent times with similar results. ***p < .001 and ****p < .0001. Scale bars were indicated in the figure.

Gene ontology (GO) analysis was used to investigate the mechanism, and we observed significant alterations (*p* < .05) in several signalling pathways, including hippo and contraction (Figure S5A), which have been proved to regulate YAP/TAZ activity.^9^, ^10^ Notably, YAP/TAZ activity and WNT4 levels significantly increased in the injured group (Figure S5B–H). Intriguingly, YAP ChIP‐seq indicated that WNT4 is a putative target of YAP/TAZ (Figure 3A,B), and YAP/TAZ knockout in SMCs decreased WNT4 levels (Figure 3C). These data suggested that YAP/TAZ transcriptional‐regulated WNT4 in SMCs. To explore the role of SMCs’ YAP/TAZ‐WNT4 signalling, we isolated the SMCs, collected their supernatant and cultured it with WT organoids. The results implied that WT SMCs supernatant increased organoid area, while YAP/TAZ cKO SMCs abolished this effect (Figure 3D,E). Meanwhile, in vivo experiments were used to confirm our results by 3.5% DSS (Figure 3F) or 10 Gy whole‐body X‐ray irradiation (Figure 3K). DSS treatment resulted in higher levels of bleeding and diarrhoea, which were exacerbated by YAP/TAZ knockout in SMCs (Figure S4I,J). HE, PAS, KI67 and CL.CASPASE3 results implied that YAP/TAZ knockout in SMCs exhibited more pronounced gut damage, compared to the DSS/IR‐treated WT group (Figures 3G,L, S5M,N and S6A,B). The levels of YAP/TAZ and WNT4 in colon SMCs or LRP5 in TA cells were elevated in response to DSS/IR treatment, but not in YAP/TAZ knockout mice (Figures 3H–J, M–O, S5K,O and S6C). Moreover, the WNT4 ELISA results found that DSS treatment increased WNT4 secretion, which was restored to baseline levels upon YAP/TAZ knockout (Figure S5L). Together, these findings suggest that the SMCs' YAP/TAZ‐WNT4 signalling cascade plays a crucial role in injured gut regeneration.

**FIGURE 3:**
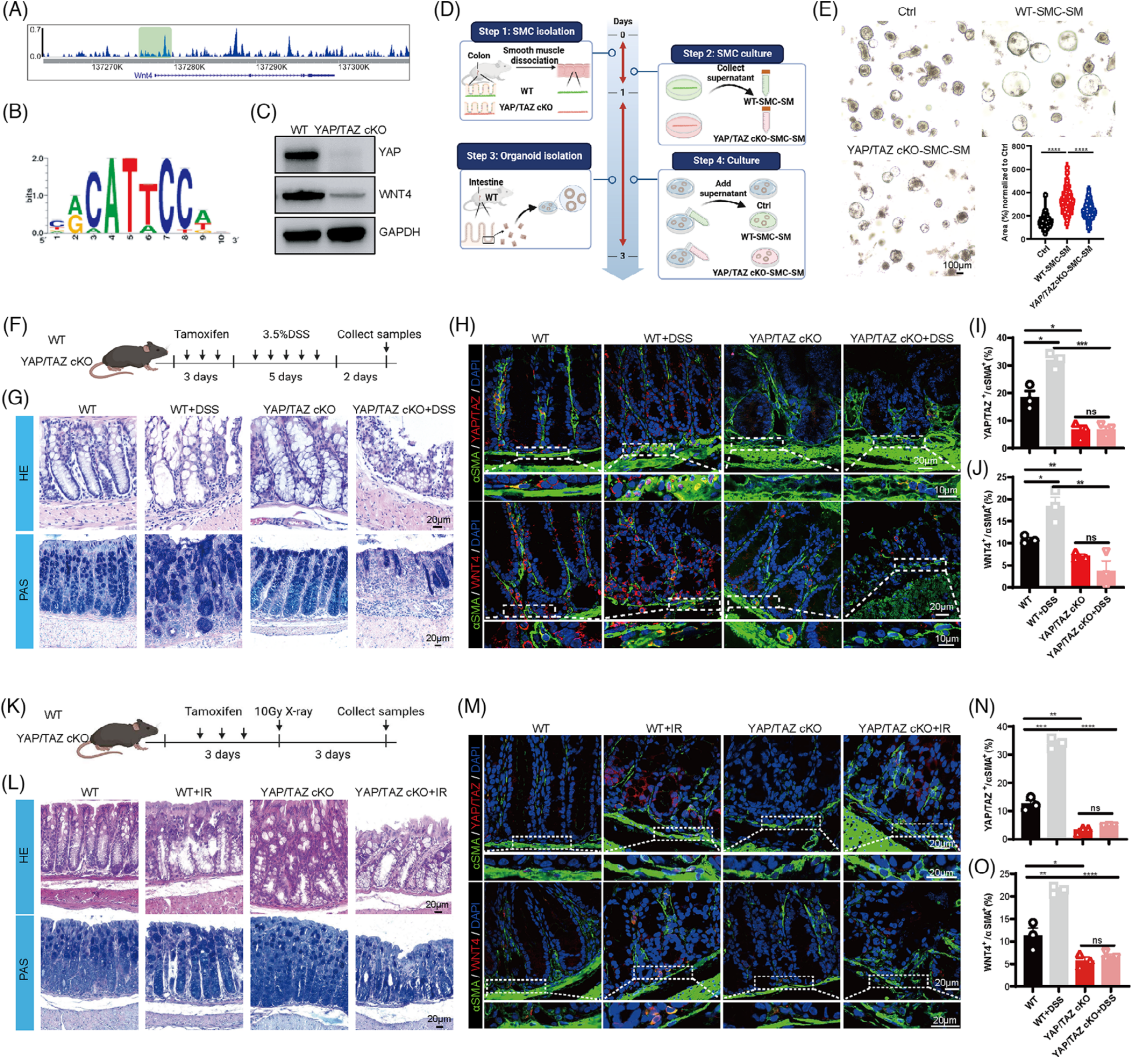
SMCs yes‐associated protein (YAP)/transcriptional coactivator with PDZ‐binding motif (TAZ) knockout decreased the gut regeneration by regulating WNT4. (A) The binding areas of YAP1 and WNT4 promoter by ChIP‐seq. (B) The binding motif of Tead on WNT4 target gene in dextran sodium sulphate (DSS) groups. (C) Western blots were used to assess the expression of Yap and WNT4 in colon smooth muscle from WT and YAP/TAZ cKO mice. (D) Schematic illustration of the experimental workflow of intestinal organoid culture. (E) Representative image and quantification of intestinal organoid cultured with supernatant of WT or YAP/TAZ cKO SM for 3 days. (F) Schematic illustration of the experimental workflow of DSS treatment. (G) Representative colon HE images (upper) and PAS images (bottom) from WT or YAP/TAZ cKO mice treated with DSS. Representative image (H, upper) and quantifications (I) of immunofluorescence images of YAP/TAZ (red), α‐smooth muscle actin (αSMA) (green) and 4',6‐diamidino‐2‐phenylindole (DAPI) (blue) from WT or YAP/TAZ cKO mice treated with DSS. Representative image (H, bottom) and quantifications (J) of immunofluorescence images of WNT4 (red), αSMA (green) and DAPI (blue) from WT or YAP/TAZ cKO mice treated with DSS. (K) Schematic illustration of the experimental workflow of X‐ray treatment. (L) Representative colon HE images (upper) and PAS images (bottom) from WT or YAP/TAZ cKO mice treated with 10 Gy X‐ray. Representative image (M, upper) and quantifications (N) of immunofluorescence images of YAP/TAZ (red), αSMA (green) and DAPI (blue) from WT or YAP/TAZ cKO mice treated with 10 Gy X‐ray. Representative image (M, bottom) and quantifications (O) of immunofluorescence images of WNT4 (red), αSMA (green) and DAPI (blue) from WT or YAP/TAZ cKO mice treated with 10 Gy X‐ray. Violin plots indicate median (middle line), 25th, 75th percentile, minima, maxima. Bar charts are presented as the mean ± SEM. The statistical analysis was calculated by two‐sides unpaired Student's *t*‐test, the confidence interval is 95%. The point represents a mouse or organoid sample. Each experiment was repeated three independent times with similar results. *p < .05, **p < .01, ***p < .001 and ****p < .0001. Scale bars were indicated in the figure.

Next, we investigated whether mechanotransduction could influence YAP/TAZ‐WNT4 signalling in SMCs. We observed a significant upregulation of extracellular stimulus, smooth muscle contraction and mechanical stimulus in SMCs (Figures 4A and S7A,B). Moreover, the levels of pMLC and YAP/TAZ activity increased in DSS group (Figure S7D–H). Furthermore, low mechanical inputs such as contraction drugs, soft extracellular matrixc (ECM), densely plated and mechanotransduction inhibitors significantly decreased the YAP/TAZ activity and WNT4 levels (Figures 4B and S7C). Tada or Adeno supernatants decreased organoid area and WNT4 secretion from SMCs compared with control (Figure 4C–F). Subsequently, we conducted in vivo experiments to validate our findings using 3.5% DSS and high mechanical inputs (Nopp and Vaso; Figure S7I–K). The results suggested that mechano‐stimulatory drugs significantly reduced the levels of CXCL1 and IL6 in comparison to the DSS‐treated WT group, but not in DSS‐treated YAP/TAZ cKO mice (Figure 4K). HE, PAS, KI67 and CL.CASPASE3 results suggested the similar outcomes (Figures 4G,H, S7L,M and O,P). Furthermore, the levels of YAP/TAZ and WNT4 in colon SMCs or LRP5 in TA cells were elevated in response to mechano‐stimulatory drugs compared to DSS‐treated WT group, but these increases were nullified in YAP/TAZ cKO mice (Figure S7I–K and N,Q).

**FIGURE 4:**
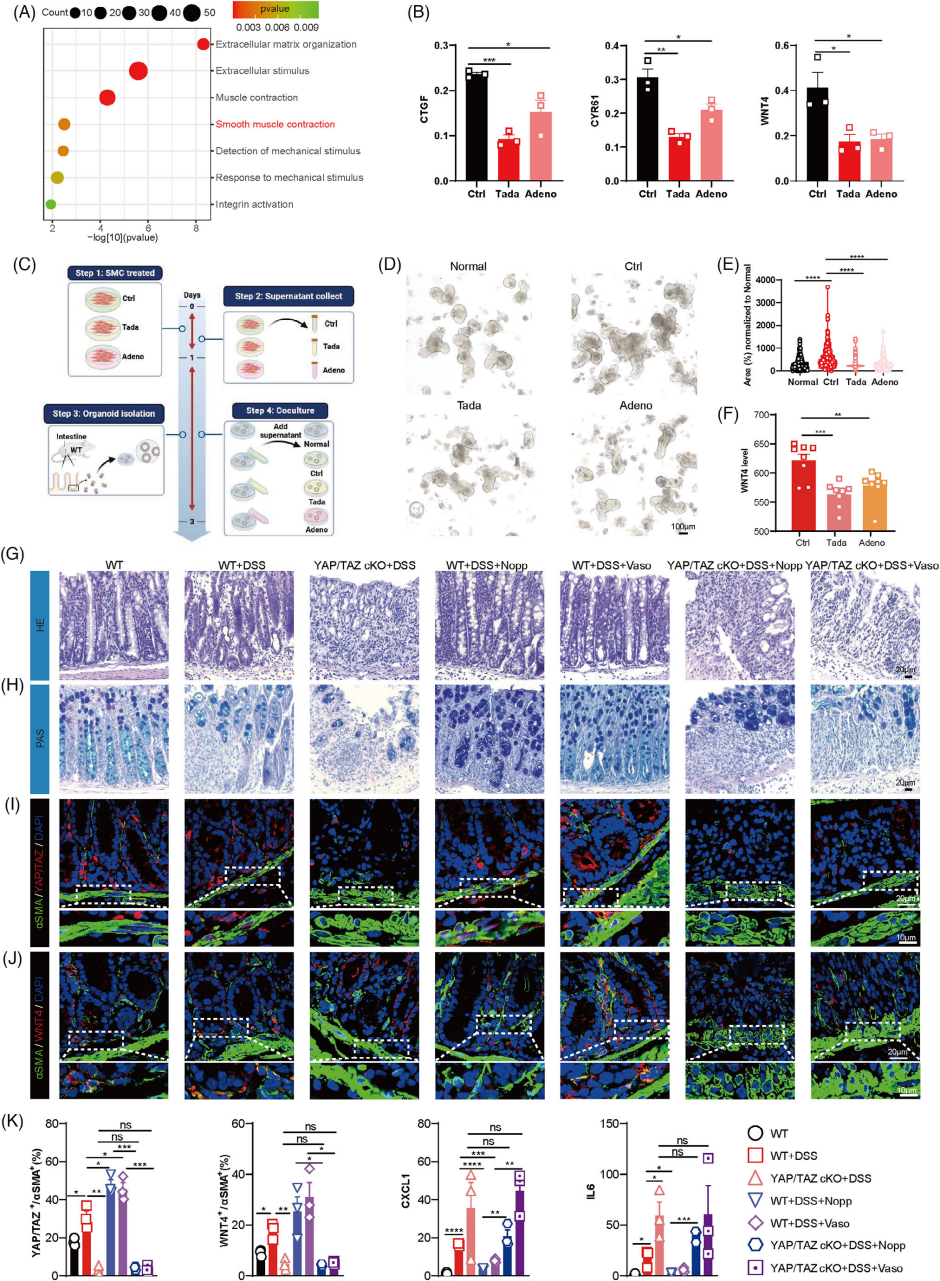
Mechanotransduction influenced the gut regeneration by yes‐associated protein (YAP)/transcriptional coactivator with PDZ‐binding motif (TAZ)‐WNT4 cascades. (A) GO analysis according to the differential expressed genes (DEGs) of SMCs in Con and dextran sodium sulphate (DSS) groups. (B) Expression of CTGF, CYR61 and WNT4 in dense plated MOVAS cells treated with Tadalafil and Adenosine. (C) Schematic illustration of the experimental workflow of intestinal organoid culture. Representative (D) and quantification (E) intestinal organoid cultured with supernatant of dense plated MOVAS cells treated with Tadalafil and Adenosine for 3 days. (F) The levels of WNT4 from the supernatant of dense plated MOVAS cells treated with Tadalafil and Adenosine. Representative colon HE images (G) and PAS images (H) from WT or YAP/TAZ cKO mice treated with DSS or contraction drugs (Nopp and Vaso are pro‐contraction drugs). Representative image (I) and quantifications (K) of immunofluorescence of YAP/TAZ (red), αSMA (green) and DAPI (blue) from WT or YAP/TAZ cKO mice treated with DSS or contraction drugs. Representative image (J) and quantifications (K) of immunofluorescence of WNT4 (red), αSMA (green) and DAPI (blue) from WT or YAP/TAZ cKO mice treated with DSS or contraction drugs. (K) Expression of CXCL1, IL6 and the quantifications of YAP/TAZ and WNT4 immunofluorescence in DSS from WT or YAP/TAZ cKO mice treated with DSS or contraction drugs. Violin plots indicate median (middle line), 25th, 75th percentile, minima, maxima. Bar charts are presented as the mean ± SEM. The statistical analysis was calculated by two‐sides unpaired Student's *t*‐test, the confidence interval is 95%. The point represents a mouse or organoid sample. Each experiment was repeated three independent times with similar results. *p < .05, **p < .01, ***p < .001 and ****p < .0001. Scale bars were indicated in the figure.

In summary, we found that mechanotransduction promoted injured gut regeneration through YAP/TAZ‐WNT4 signalling axis in crosstalk between SMCs and TA cells.

## AUTHOR CONTRIBUTIONS

Conceptualisation: Mintao Ji, Shuai Dong, Zhenke Wen, Guangming Zhou and Lei Chang; Methodology: Mintao Ji, Shuai Dong, Haisheng Liang, Shuangshuang Lu, Chenyu Luo, Haimeng Zheng, Yinyin Shu, Zhisen Zhang and Xiaoni Jin; Investigation: Mintao Ji, Shuai Dong, Haisheng Liang, Yuhan Guo, Hong Zhang and Kai Kang; Visualisation: Mintao Ji, Shuangshuang Lu, Yiping Lin, Yuhong Wang, Sha Huang, Xiaobing Fu and Lei Chang; Funding acquisition: Mintao Ji, Guangming Zhou and Lei Chang; Project administration: Mintao Ji, Guangming Zhou and Lei Chang; Supervision: Zhenke Wen, Guangming Zhou and Lei Chang; Writing‐original draft: Mintao Ji, Guangming Zhou and Lei Chang; Writing‐review & editing: Shuai Dong, Hanna Lucie Sladitschek‐Martens, Zhenke Wen, Guangming Zhou and Lei Chang.

## CONFLICT OF INTEREST STATEMENT

The authors declare no conflicts of interest.

## ETHICS STATEMENT

The study was approved by the ethics committee of Soochow University. All studies were conducted in accordance with the ARRIVE (Animal Research: Reporting of In Vivo Experiments) guidelines and the Helsinki declaration.

## Supporting information

Supporting Information

Supporting Information

Supporting Information

Supporting Information

Supporting Information

Supporting Information

Supporting Information

Supporting Information

Supporting Information

## Data Availability

All images and raw data for statistical analysis in this study are available from the corresponding author upon reasonable request.
