# Supplementary figures and images for: Mechano‐YAP/TAZ‐regulated smooth muscle cells are an important source of Wnt signalling for gut regeneration
Source: Clin Transl Med. 2024 Aug 16;14(8):e70005. doi: 10.1002/ctm2.70005 (PMC11329746; doi:10.1002/ctm2.70005)

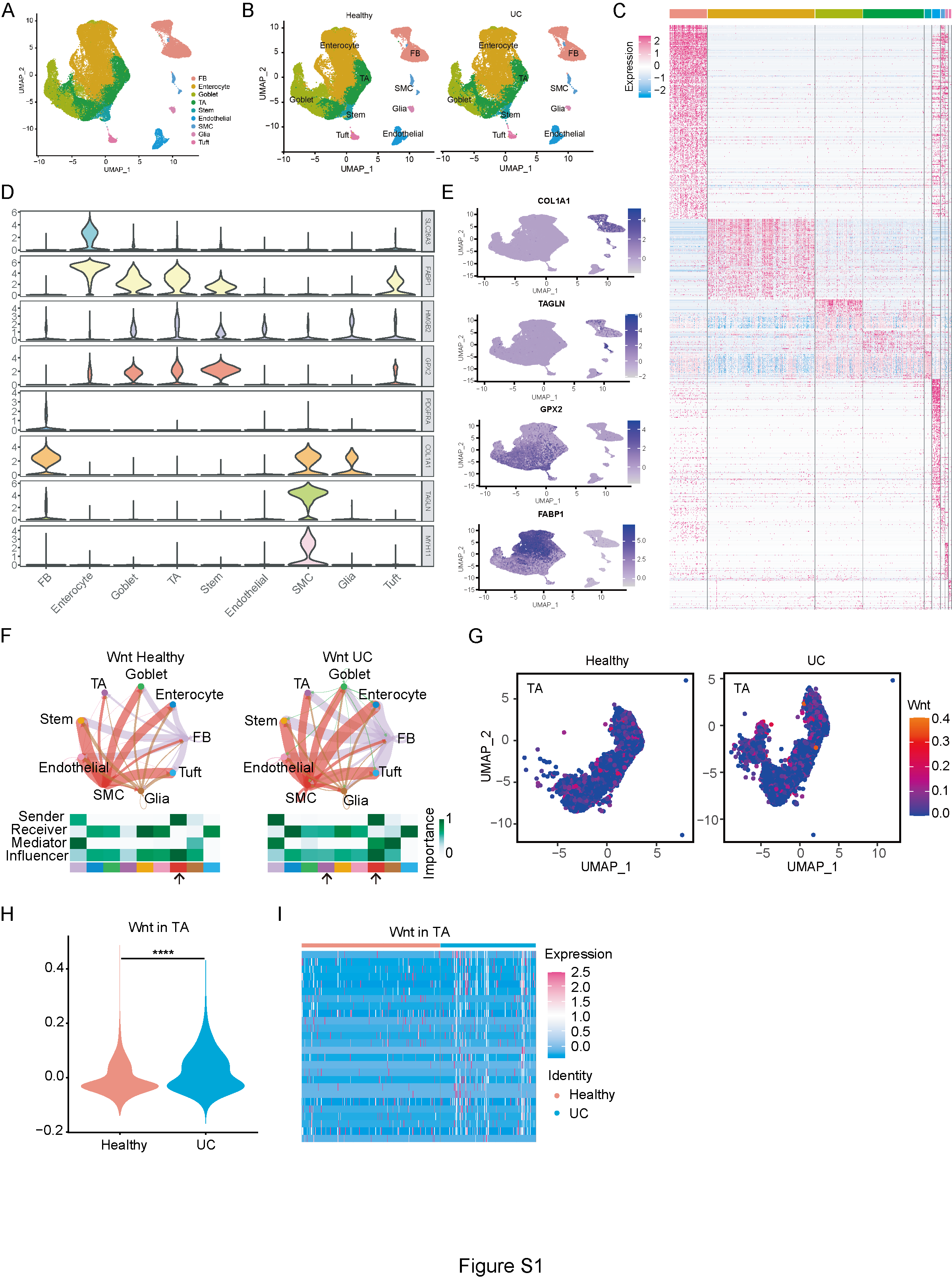

Supplement: Supplementary file 1 — Supporting Information [file CTM2-14-e70005-s003.tif]

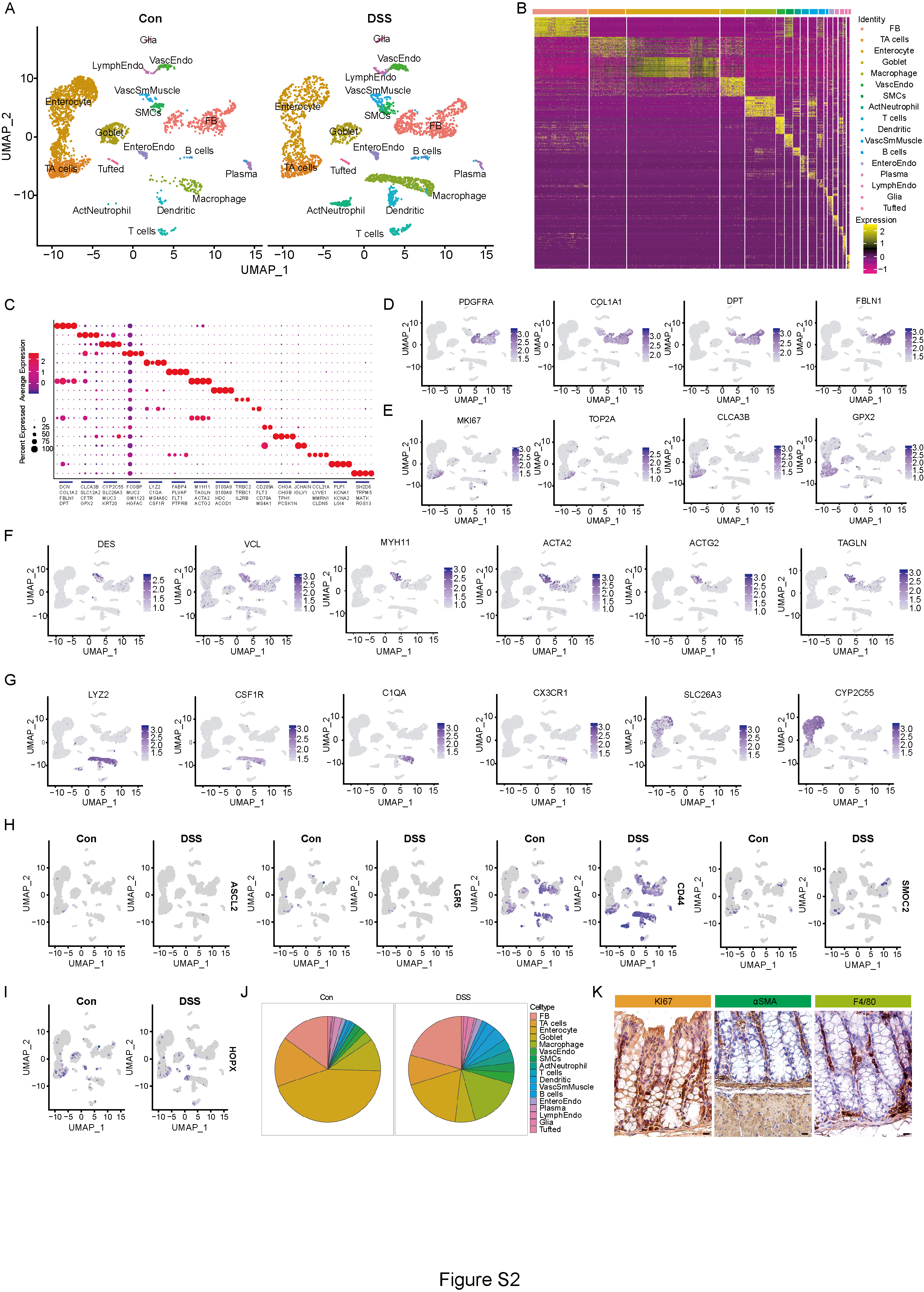

Supplement: Supplementary file 2 — Supporting Information [file CTM2-14-e70005-s005.tif]

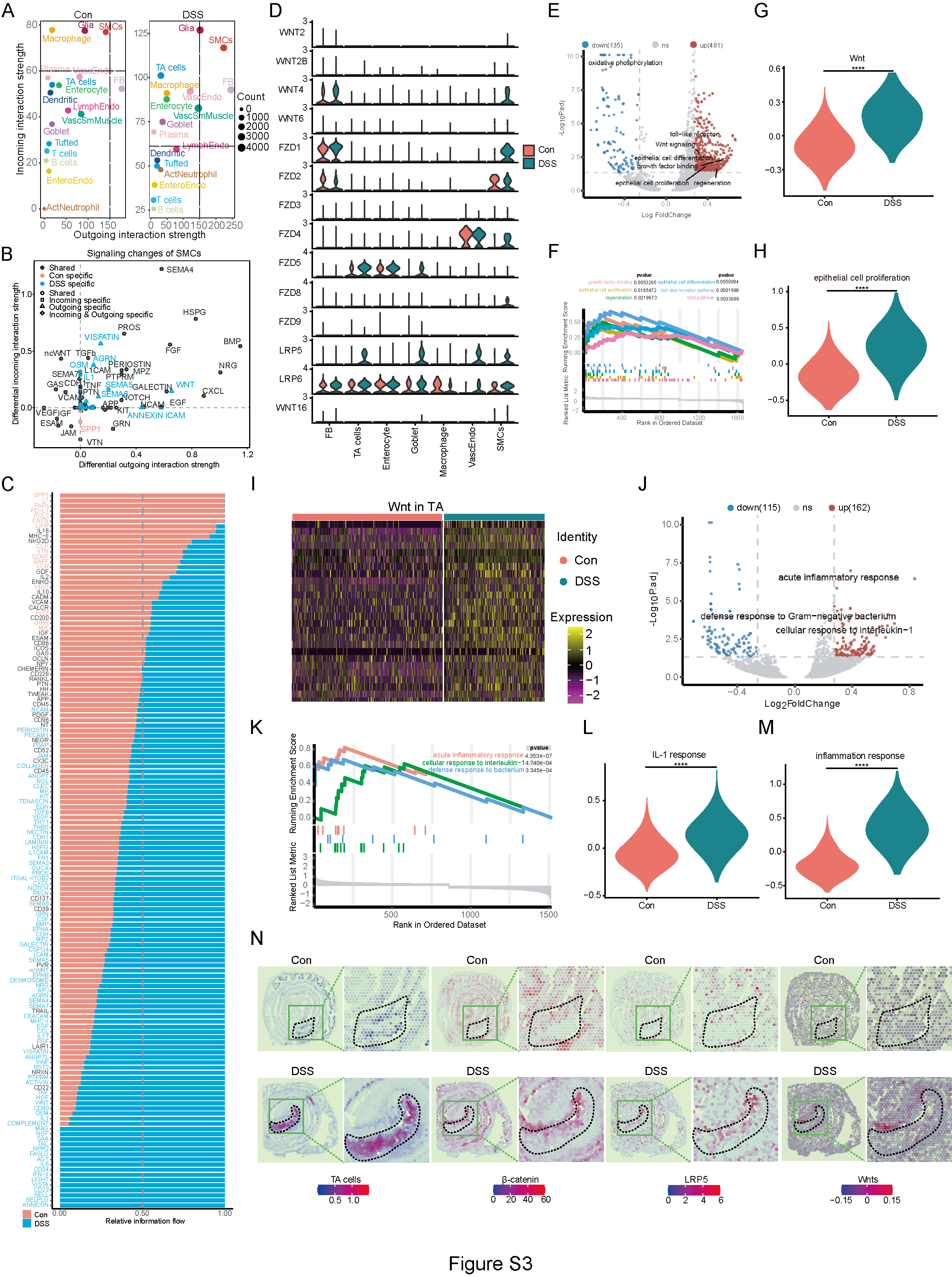

Supplement: Supplementary file 3 — Supporting Information [file CTM2-14-e70005-s008.tif]

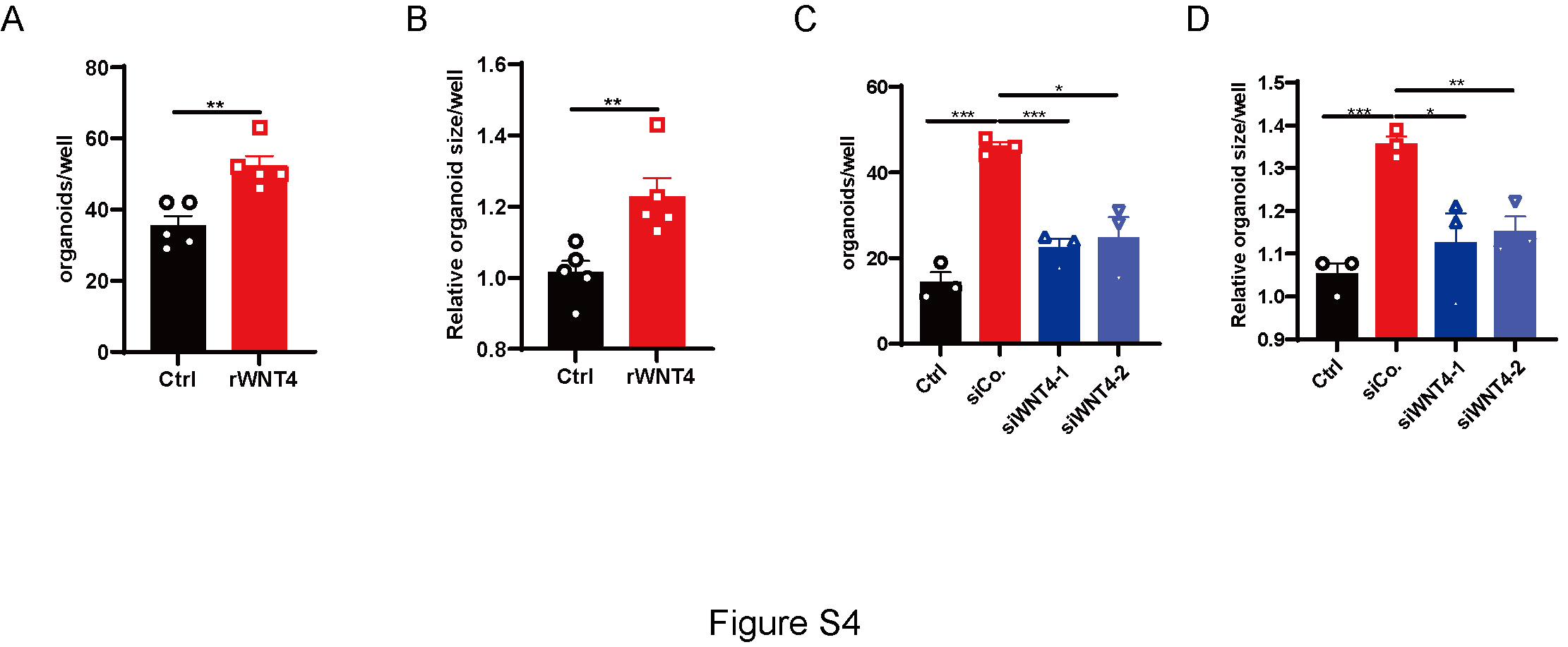

Supplement: Supplementary file 4 — Supporting Information [file CTM2-14-e70005-s006.tif]

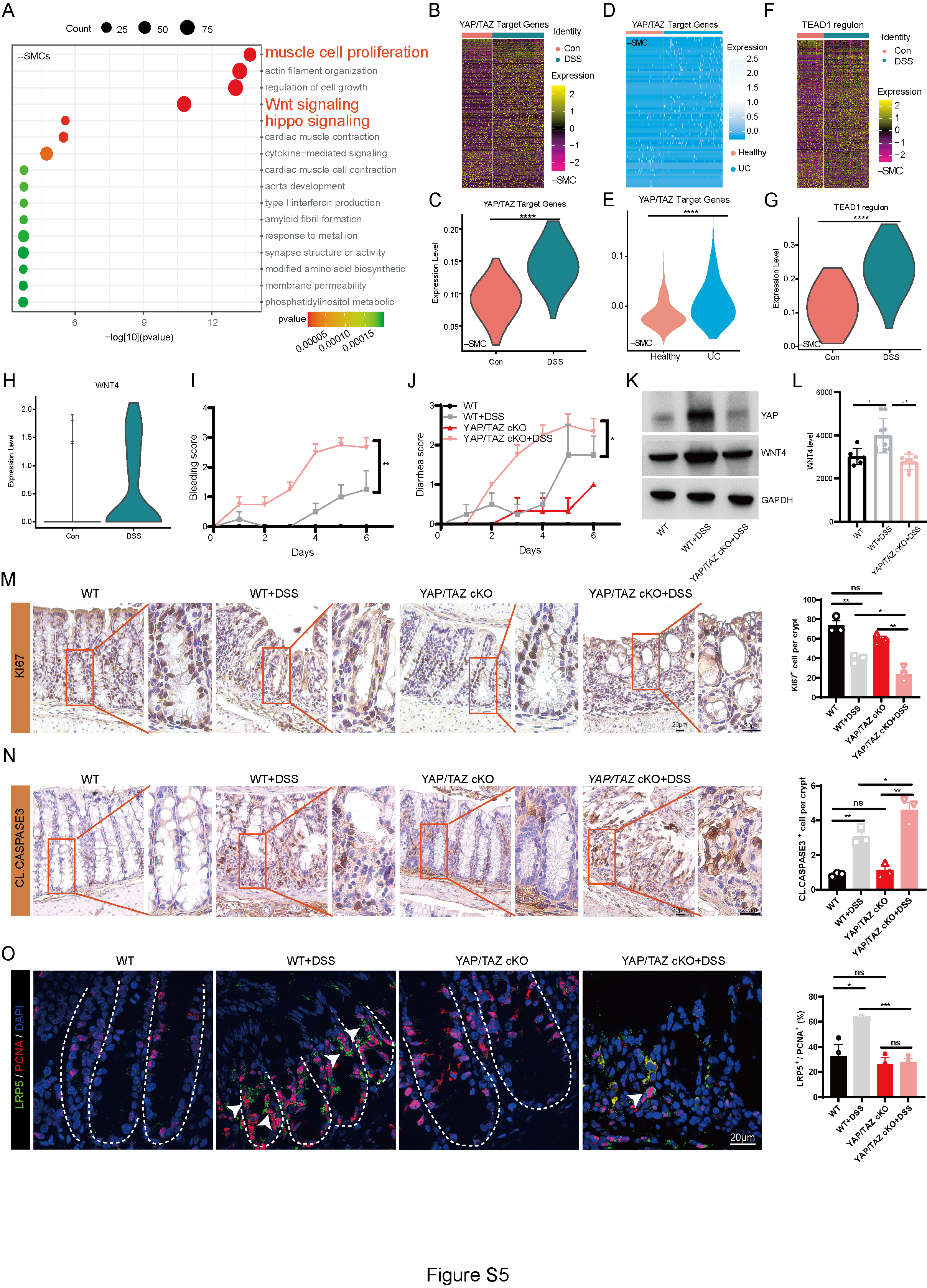

Supplement: Supplementary file 5 — Supporting Information [file CTM2-14-e70005-s004.tif]

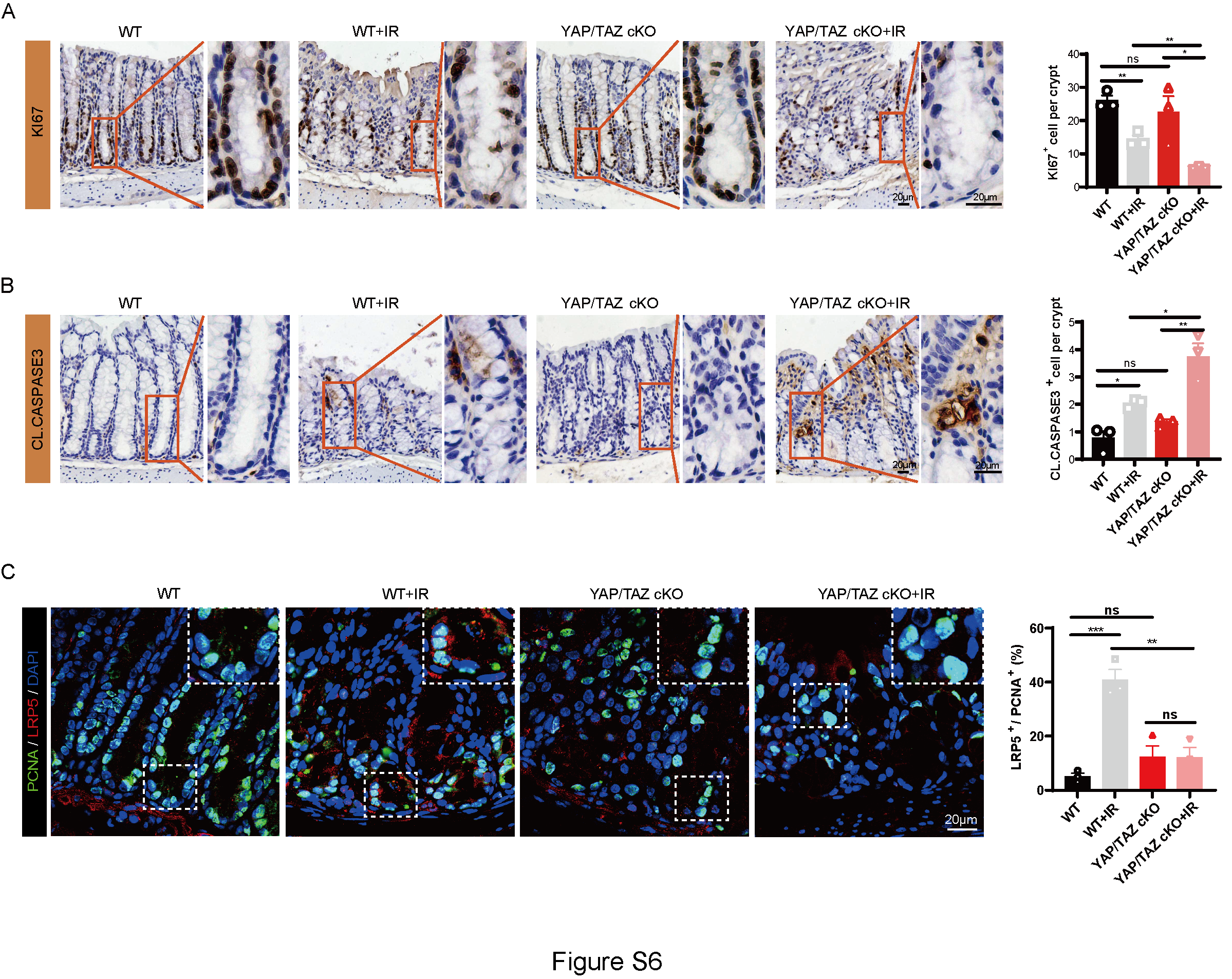

Supplement: Supplementary file 6 — Supporting Information [file CTM2-14-e70005-s007.tif]
