# Supplementary material for: Mechano‐YAP/TAZ‐regulated smooth muscle cells are an important source of Wnt signalling for gut regeneration
Source: Clin Transl Med. 2024 Aug 16;14(8):e70005. doi: 10.1002/ctm2.70005 (PMC11329746; doi:10.1002/ctm2.70005)
Supplement: Supplementary file 9 — Supporting Information [file CTM2-14-e70005-s002.docx]

Supplementary Materials for

Mechano-YAP/TAZ regulated smooth muscle cells are an important source of Wnt signaling for gut regeneration

Mintao Ji^1^†, Shuai Dong^1^†, Shuangshuang Lu^1^, Haisheng Liang^1^, Yiping Lin^1^, Chenyu Luo^1^, Haimeng Zheng^1^, Yinyin Shu^1^, Zhisen Zhang^1^, Xiaoni Jin^1^, Yuhan Guo^1^, Kai Kang^1^, Hong Zhang^2^, Yuhong Wang^2^, ﻿Hanna Lucie Sladitschek-Martens^3^, Sha Huang^4^, Xiaobing Fu^4^, Guangming Zhou^1^*, Zhenke Wen^5^*, Lei Chang^1^*

*Corresponding Email: Lei Chang: [changlei@suda.edu.cn](mailto:changlei@suda.edu.cn); Guangming Zhou: gmzhou@suda.edu.cn; and Zhenke Wen: zkwen@suda.edu.cn.

†These authors contributed equally to this work.

This PDF file includes:

Figure S1-7

Table S1-3

**Supplementary Fig. 1. Global expression profiling of human colon by scRNA-seq.**

(**A**) Uniform Manifold Approximation and Projection (UMAP) plots illustrate the distribution of cluster cells. The cells are color-coded based on their respective types or states. (**B**) UMAP plots of cluster cells from healthy and UC human samples. (**C**) Heatmap of the DEGs in each major cluster. (**D**) Violin plots highlight the expression patterns of characteristic genes within the cell clusters. (**E**) Expression patterns of the following marker genes for each cluster are projected on the UMAP plot of all cells: COL1A1 (FB), GPX2 (TA), TAGLN (SMC), FABP1 (Enterocyte). (**F**) Inferred signaling networks for Wnt pathway in healthy and UC groups. Circle plots are shown on the top. Cell clusters that participate in signaling are annotated. Heatmaps showing the relative importance of each cell cluster as signal sender or receiver are shown at the bottom. (**G**) Representative AUC levels of Wnt signaling pathways target genes in healthy and UC groups TA cells. (**H**) Quantification of AUC levels of Wnt signaling pathways target genes in healthy and UC groups TA cells. (**I**) Heatmap of Wnt signaling pathways target genes from healthy and UC groups in TA cells. Violin plots indicate median (middle line), 25th, 75th percentile, minima, maxima. The statistical analysis was calculated by two-sides unpaired Student’s *t*-test, the confidence interval is 95%. *****p* < 0.0001.

**Supplementary Fig. 2.** **Global expression profiling of mice colon cells in Con and DSS samples by scRNA-seq.**

(**A**) Uniform Manifold Approximation and Projection (UMAP) plots of cluster cells from two Con and three DSS mice. Cells are colored according to their types or state. (**B-C**) Heatmap depicts the DEGs in each major cluster, with the top 4 DEGs are visualized as a bubble diagram. Expression patterns of the following marker genes for each cluster are projected on the UMAP plot of all cells: Pdgfra, Col1a1, Dpt and Fbln1 (FB, **D**), Des, Vcl, Myh11, Acta2, Actg2 and Tagln (SMCs, **F**), Mki67, Top2a, Clca3b and Gpx2 (TA cells, **E**), Lyz2, Csf1r, C1qa and Cx3cr1 (Macrophage, **G**), Slc26a3 and Cyp2c55 (Enterocyte, **G**). (**H-I**) Expression patterns of the marker genes for TA cells, including ASCL2, LGR5, CD44, SMOC2 and HOPX. (**J**) The percentage of each cell cluster the Control (Con) and DSS-treated (DSS) groups is presented. (**K**) Representative immunohistochemistry images of mouse colon tissue demonstrate the presence of TA cells (KI67), SMCs (αSMA) and Macrophage (F4/80). Scale bars, 10 µm.

**Supplementary Fig. 3.** **SMCs enhance TA cells' Wnt activity via WNT4 ligand-receptor interactions.**

(**A**) Visualization of overall incoming and outgoing communication strength based on receptor and ligand expression in Con (left) and DSS samples (right). (**B**) Incoming and outgoing interaction signaling pathways among SMCs are depicted for both the Control (Con) and DSS-treated (DSS) groups. (**C**) The relative information flow of signaling pathways is illustrated for the Con and DSS groups. (**D**) The levels of Wnt ligand-receptor pairs from SMCs to TA cells in Con and DSS groups. (**E**) Volcano plot visualizes the significantly changed signaling pathways between Con and DSS groups specifically in TA cells. (**F**) GSEA analysis reveals the signaling pathways that exhibit significant changes between the Con and DSS groups in TA cells. (**G-H**) Target genes expression of Wnt and Cell proliferation signaling pathways from Con and DSS groups in TA cells. (**I**) Heatmap of Wnt signaling pathways target genes in TA cells of Con and DSS groups. (**J**) Volcano plot displays the significantly changed signaling pathways between the Con and DSS groups in Enterocyte cells. (**K**) GSEA analysis identifies the signaling pathways that show significant changes between the Con and DSS groups in Enterocyte cells. (**L-M**) Target genes expression of Il1 and Inflammation response signaling pathways from Con and DSS groups in Enterocyte cells. (**N**) Spatial distribution analysis of TA cells, the expression of β-catenin, LRP5 and Wnts (Black dashed circle) from the Con and DSS groups. Violin plots indicate median (middle line), 25th, 75th percentile, minima, maxima. The statistical analysis was calculated by two-sides unpaired Student’s *t*-test, the confidence interval is 95%. *****p* < 0.0001.

**Supplementary Fig. 4.** **SMCs WNT4 knockout decreased organoid growth.**

(**A-B**) Quantification of numbers and relative size of colon organoid growth in control and rWNT4 groups for 3 days. (**C-D**) Quantification of numbers and relative size of colon organoid cocultured with supernatant of control, siCo., siWNT4-1 and siWNT4-2 groups for 3 days. Bar charts are presented as the mean ± sem. The statistical analysis was calculated by two-sides unpaired Student’s *t*-test, the confidence interval is 95%. The point represents an organoid sample. Each experiment was repeated three independent times with similar results. **p* < 0.05, ***p* < 0.01, ****p* < 0.001.

**Supplementary Fig. 5.** **SMCs YAP/TAZ knockout decreased injured gut regeneration induced by DSS.**

(**A**) GO analysis according to the DEGs of SMCs in Con and DSS groups. (**B**) Heatmap of YAP/TAZ target genes in Con and DSS SMCs. (**C**) Quantification of AUC levels of YAP/TAZ target genes in Con and DSS SMCs. (**D**) Heatmap of YAP/TAZ target genes in healthy and UC SMCs. (**E**) Quantification of AUC levels of YAP/TAZ target genes in healthy and UC SMCs. (**F**) Heatmap of TEAD1 regulon regulated genes in Con and DSS SMCs. (**G**) Quantification of AUC levels of TEAD1 regulon regulated genes in Con and DSS SMCs. (**H**) The levels of WNT4 in Con and DSS SMCs. Quantifications of the bleeding score (**I**) and diarrhea score (**J**) in WT or YAP/TAZ cKO mice treated with DSS. (**K**) Western blots were used to assess the expression of YAP and WNT4 in colon smooth muscle from WT and YAP/TAZ cKO mice. (**L**) The levels of WNT4 from the supernatant of WT or YAP/TAZ cKO SM treated with DSS. (**M**) Representative image (left) and quantifications (right) of KI67 colon immunohistochemistry from WT or YAP/TAZ cKO mice treated with DSS. (**N**) Representative image (left) and quantifications (right) of CL.CASPASE3 colon immunohistochemistry from WT or YAP/TAZ cKO mice treated with DSS. (**O**) Representative image and quantifications of immunofluorescence images of LRP5 (green), PCNA (red) and DAPI (blue) from WT or YAP/TAZ cKO mice treated with DSS. Violin plots indicate median (middle line), 25th, 75th percentile, minima, maxima. Bar charts are presented as the mean ± sem. The statistical analysis was calculated by two-sides unpaired Student’s *t*-test, the confidence interval is 95%. The point represents a mouse sample. Each experiment was repeated three independent times with similar results. **p* < 0.05, ***p* < 0.01, ****p* < 0.001, *****p* < 0.0001. Scale bars, 20 µm.

**Supplementary Fig. 6.** **SMCs YAP/TAZ knockout decreased injured gut regeneration induced by X-ray irradiation.**

(**A**) Representative image (left) and quantifications (right) of KI67 colon immunohistochemistry from WT or YAP/TAZ cKO mice treated with 10 Gy X-ray. (**B**) Representative image (left) and quantifications (right) of CL.CASPASE3 colon immunohistochemistry from WT or YAP/TAZ cKO mice treated with 10 Gy X-ray. (**C**) Representative image (left) and quantifications (right) of immunofluorescence images of LRP5 (red), PCNA (green) and DAPI (blue) from WT or YAP/TAZ cKO mice treated with 10 Gy X-ray. Bar charts are presented as the mean ± sem. The statistical analysis was calculated by two-sides unpaired Student’s *t*-test, the confidence interval is 95%. The point represents a mouse sample. Each experiment was repeated three independent times with similar results. **p* < 0.05, ***p* < 0.01, ****p* < 0.001. Scale bars, 20 µm.

**Supplementary Fig. 7. Mechanotransduction influenced the gut regeneration by YAP/TAZ-WNT4 cascades.**

(**A**) The changes of smooth muscle contraction signaling pathway by GSEA analyze between Con and DSS groups in SMCs. (**B**) Heatmap of Smooth muscle contraction signaling pathway target genes from Con and DSS groups in SMCs. (**C**) Expression of CTGF, CYR61 and WNT4 in MOVAS cells plated in stiff or soft mechanical environments, in Sparse or Dense state, or treated with mechanical inhibitors, such as Ble, Ceri, Y27632 and Lat.A. (**D**) Representative immunohistochemistry of pMLC from Con and DSS colon groups. (**E**) Representative image of immunofluorescence images of YAP/TAZ (red), αSMA (green) and DAPI (blue) from Con and DSS groups. Spatial expression of MYH11 (**F**), TEAD1 (**G**) and TAZ (**H**) in SMCs (Red dashed circle) from Con and DSS groups. (**I**) Schematic illustration of the experimental workflow of DSS treatment. (**J**) Quantifications of colon length in WT or YAP/TAZ cKO mice treated with DSS or mechano-related drugs. (**K**) Representative images of colon length in WT or YAP/TAZ cKO mice treated with DSS or mechano-related drugs. (**L**) Quantifications of colon KI67 immunohistochemistry from WT or YAP/TAZ cKO mice treated with DSS or contraction drugs. (**M**) Quantifications of colon CL.CASPASE3 immunohistochemistry from WT or YAP/TAZ cKO mice treated with DSS or contraction drugs. (**N**) Quantifications of immunofluorescence images of LRP5 (green), PCNA (blue) from WT or YAP/TAZ cKO mice treated with DSS or mechano-related drugs. Representative image of KI67 (**O**) and CL.CASPASE3 (**P**) colon immunohistochemistry from WT or YAP/TAZ cKO mice treated with DSS or contraction drugs. (**Q**) Representative images of immunofluorescence images of LRP5 (green), PCNA (blue) from WT or YAP/TAZ cKO mice treated with DSS or mechano-related drugs. Bar charts are presented as the mean ± sem. The statistical analysis was calculated by two-sides unpaired Student’s *t*-test, the confidence interval is 95%. The point represents a mouse sample. Each experiment was repeated three independent times with similar results. **p* < 0.05, ***p* < 0.01, ****p* < 0.001, *****p* < 0.0001. Scale bars, 20 µm.
